# Failure Rate of Dental Implants in the Esthetic Zone: A Systematic Review and Meta-Analysis

**DOI:** 10.7759/cureus.65506

**Published:** 2024-07-27

**Authors:** Manar Alzahrani, Sondus Bakhreibah, Nada Alharbi, Lama Alamoudi, Seba Halloul, Sara Alamoudi, Raghad Alharthi, Salem Baghdadi, Ahmed Alamoudi

**Affiliations:** 1 General Dentistry, King Abdulaziz University, Jeddah, SAU; 2 Oral Biology, King Abdulaziz University, Jeddah, SAU

**Keywords:** esthetic zone, socket-shield, failure rate, dental implant, implant

## Abstract

The present systematic review and meta-analysis has systematically reviewed and analyzed dental implant failure for the implants placed in the esthetic zone. An electronic database search was performed in PubMed and ScienceDirect, including a manual search through the references using appropriate keywords and the PICO (population, intervention, control, and outcomes) format for the inclusion criteria. A total of 11 relevant articles were included. The quality of the randomized controlled trial (*RCT*) studies was assessed using the Cochrane Risk of Bias tool while the quality of non-randomized studies was assessed using the Newcastle Ottawa scale. Of the 11 articles included, 5 were RCTs, and 6 were non-randomized. The overall failure rate was found to be 2% (95% CI; 0.00-0.03%). The percentage marginal bone loss was estimated to be 1% (95% CI; 0.00 - 0.02%) and the mean and proportion pink esthetic scores were approximately 11.75 (0.43%) with 2% mid-facial soft tissue recession and the mesial and distal papillary recession was 0.02% and 0.01%, respectively. Based on this systematic review and meta-analysis, the rate of dental implant failure for implant placement in the esthetic zone was minimal. In addition, 1% proportional marginal bone loss and moderately high esthetic scores were found.

## Introduction and background

When seeking treatment for tooth loss, patients have a variety of options available [1]. Clinicians facilitate decision-making by sharing information about the risks and benefits of these options [1]. Dental implant-supported prostheses have expanded treatment choices, improving patient-dentist discussions and treatment quality [1,2]. Dental implants are an excellent option for replacing missing teeth, showing an 82.9% success rate over 16 years when appropriate factors are considered [3]. Implant failure risks, influenced by age, sex, smoking, implant site, bone quality, and chronic diseases, should be monitored [4-6].

Osseointegration, the anchorage of the implant to bone, is crucial for long-term success, though failures can still occur and require removal [7,8]. Implant failure can be biological, mechanical, iatrogenic, or due to inadequate adaptation [9-11]. Rehabilitation with implants is challenging, especially in the esthetic zone, where patients expect both functional and aesthetic results [12]. Bone and soft tissue deficiencies at the implant site significantly impact outcomes [12,13]. Techniques like immediate provisional restorations and custom healing abutments help achieve natural-looking results [12-14]. Alveolar ridge preservation involves grafting materials into a tooth socket post-extraction, using various techniques like guided bone regeneration, connective tissue grafting, and partial extraction therapy (socket shield and pontic shield) [15,16]. Root submergence, especially in the anterior maxillary region, has evolved with the socket shield technique introduced in 2007, allowing immediate implant placement [16]. This minimally invasive technique has been modified to reduce complications and improve outcomes, especially with the use of 3D imaging/CBCT for planning [17-19].

In this systematic review and meta-analysis, studies were systematically reviewed and analyzed dental implant failure placed in the esthetic zone.

## Review

Research methodology

Reporting Guidelines

This systematic review followed Preferred Reporting Item for Systematic Review (PRISMA) guidelines [20]. The methodology process was in stages. The first stage was based on inclusion and exclusion criteria, the second and third stages were information source and search strategy & study selection and quality assessment of the included studies. The last stage was the extraction of data of interest from the included studies, and then, the synthesis of results.

Research Question

To achieve the objective of the research, the research question was formulated: In adults or children with tooth caries or other dental diseases undergoing dental implants, what is the overall failure rate, success rate, and other esthetic outcomes for dental implants in the esthetic zone?

Database Search and Strategy

The search was performed using two electronic databases: PubMed, and ScienceDirect. The scope of the systematic review was based on the inclusion and exclusion criteria. We started by forming two or more search strings to form a keyword using the Boolean operator ”OR, AND”. Keywords were searched within the title, abstract, and link. A high number of results were returned, but we ensured that the most relevant studies to the study topic were obtained by manually selecting the relevant studies from the database results. The search strings and MeSH (Medical Subject Headings) that were used to search for articles are: ((“Dental Implant failure” OR “Endosseous” OR “Replacing missing teeth” OR “Hard tissue bone” OR “Soft tissue” OR “Implant position” AND “Maxillary” OR “canines” OR “maxilla” AND “Esthetic zone” AND “Socket-Shield Techniques” OR “Conventional Techniques”)).

Eligibility Criteria

The eligibility criteria for selection were based on the research question, studies published between 2000 and 2023, studies published in the English language, and studies focused on dental implants placed in the esthetic zone. The study designs included were prospective, retrospective, and randomized controlled studies. 

*Study Selection Process* 

Following the application of the keywords to the databases, 402 eligible articles were generated from the databases. The search was limited to publications from 2000 to 2023 to gather all the latest publications in the field of interest and include them in the review. All articles were screened thoroughly by two reviewers (AA, MA). At first, the reviewers screened through the abstracts and titles of the articles to select articles that were deemed fit to be eligible for the research using the inclusion criteria. Duplicate studies were removed using reference management software (EndNote), and studies that did not provide enough information when going through the abstract were removed. The second phase of screening was conducted by going through the full text of the remaining studies and irrelevant studies were removed. A PRISMA flowchart was subsequently generated for the final selection of studies to be included.

Data Extraction and Study Outcome

The following information was extracted into a pre-defined Microsoft Excel sheet (Microsoft Corporation, Redmond, WA, US). The author's first name, year of publication, country of publication, study design (randomized controlled trial and prospective, retrospective, and case studies), number of patients, age, gender, failure rate, number of bone loss, success rate, and techniques used.

*Quality Assessment* 

The Cochrane Handbook for Systematic Reviews of Interventions was followed and particularly focused on random-sequence generation, allocation concealment, blinding, outcome assessment, and selective reporting of selected studies [21]. For RCTs, we used the Cochrane risk of bias (ROB) assessment tool, which is sufficiently illustrated in the Cochrane Handbook of Systematic Reviews of Interventions version 6.0 [21]. This tool can detect five types of biases: performance bias, selection bias, detection bias, reporting bias, and attrition bias. Included RCTs could be thought to be of high, unclear, or low bias source based on these domains. For non-randomized studies, we used the Newcastle-Ottawa scale (NOS) for assessing bias sources [22]. This tool screens for the selection of exposed and non-exposed participants, the comparability between study participants, the adequacy of the follow-up period, and the clarity of the definition of intended outcomes.

Data Evaluation and Analysis

To analyze the data extracted from the included study. Two independent authors performed the analysis using RevMan software version 5.4 (Review Manager (RevMan) [Computer program]. Version 5.4. The Cochrane Collaboration, 2020). A random effect meta-analysis was performed using odds ratio as the effect size. The heterogeneity of the studies was assessed and a 95% Confidence Interval was chosen with a 5% level of significance. The final results of the meta-analysis were displayed using a Forest plot, and publication bias was measured using the Eager test and displayed using a funnel plot.

Results 

*Study Selection* 

**Figure 1:**
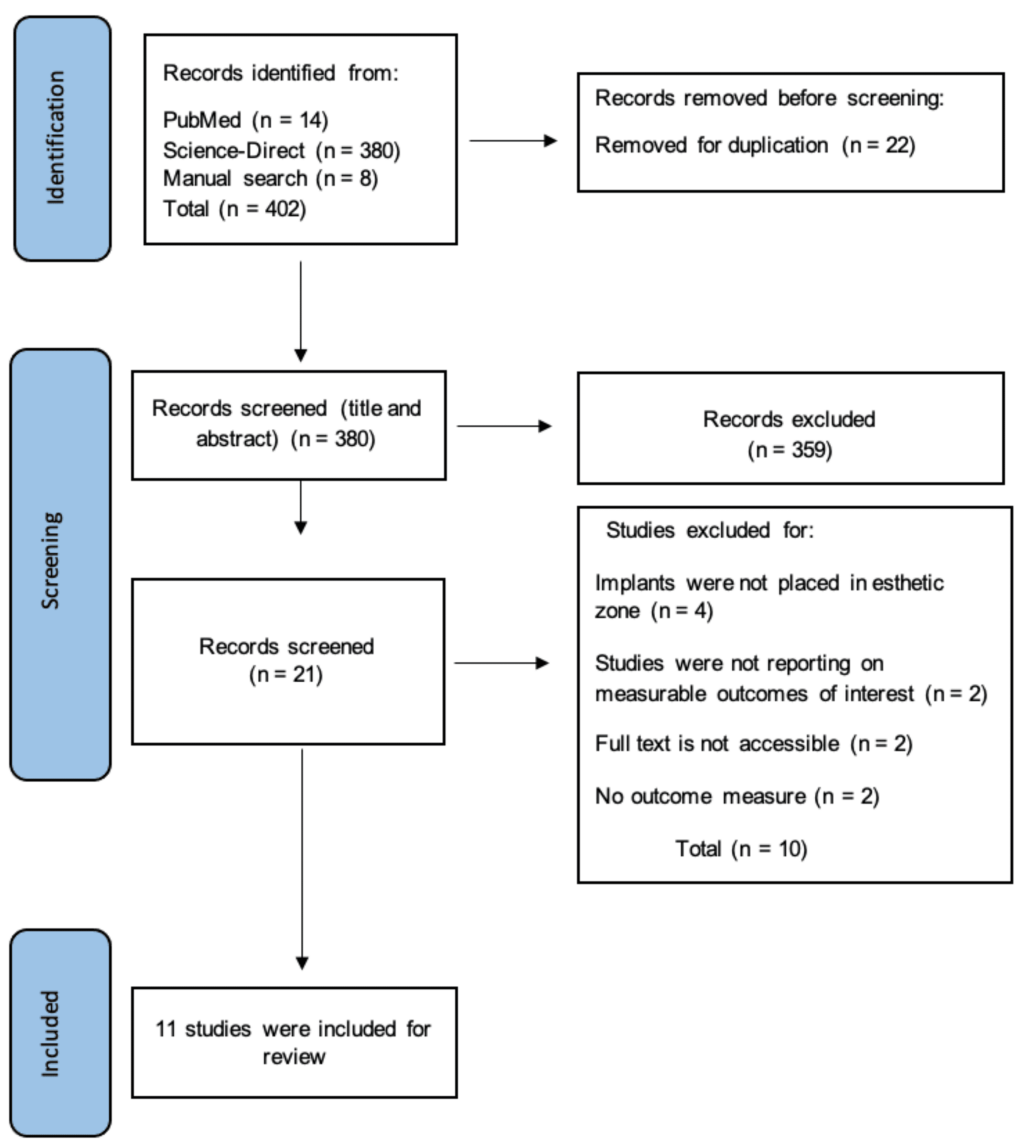
PRISMA flow chart PRISMA: Preferred Reporting Items for Systematic Review

A total of 402 articles were generated from the databases (14 articles from PubMed, 380 articles from ScienceDirect, and 8 articles through a manual search. After reviewing the reference list, the found articles were screened based on the inclusion criteria, and two additional articles were obtained. Eligible criteria following the PICO (population, intervention, control, and outcomes) format were applied to all articles and 21 articles were identified as potential articles after screening through the abstracts and title. The second stage of screening was full-text screening; the reviewers went through the full text of the 21 articles thoroughly, and 11 articles met the inclusion criteria and were included in the systematic review. See the PRISMA flow chart in Figure 1.

Results of the Quality Assessment

**Figure 2:**
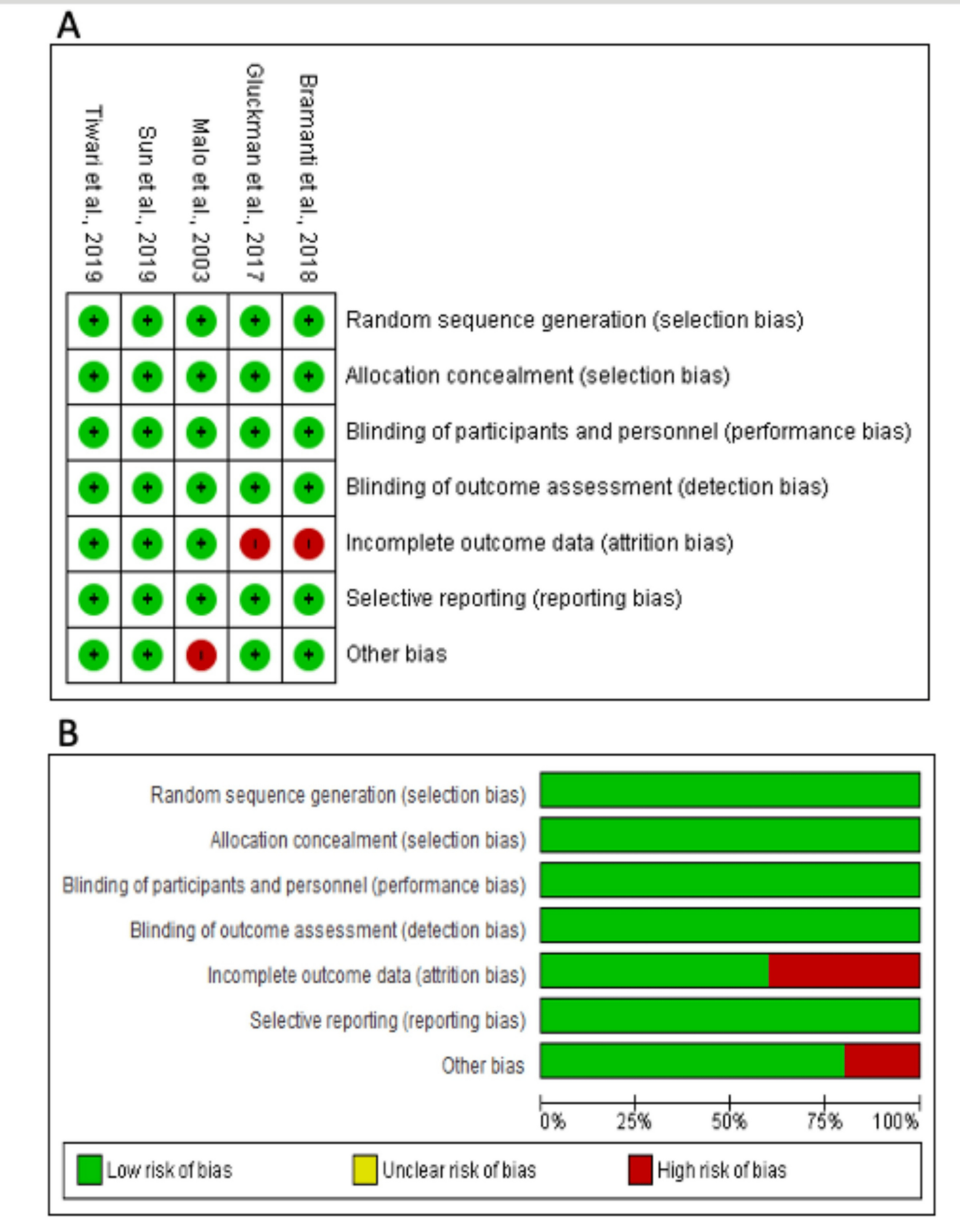
Result of the quality assessment using ROB 1.0 A) Quality assessment of the randomized clinical trial studies; B) Summary of quality assessment from the risk of bias (ROB) tool References [23,24,26,31,33]

Five RCTs were assessed for quality using ROB [23-33]. The two RCTs had a high risk of bias under the incomplete data outcome domain [26,31]. Other biases, such as the sample size for each group, were not specified in one study (Figure 2) [32]. For non-randomized studies, Table 1 shows the quality assessment of the results [25,27-30,32]. The non-randomized studies had the highest score of 7 with the lowest score of 6 out of the total score of 7, which is an indication of a good assessment with a low risk of bias (Table 1).

**Table 1:** Results of the quality assessment of included studies using the Newcastle Ottawa scale

Studies	Selection				Comparability	Outcome		Score
	Representativeness of the exposed cohort	Selection of the non-exposed cohort	Ascertainment of exposure	Precision of exposure	Confounding controlled	Outcome assessment	Adequate follow-up	Total
Siormpa, (2014) [25]	*	*	*	*	*	*	*	7
Amir Alireza, (2015) [27]	*	*	-	*	*	*	*	6
Derouck et al., (2008) [28]	*	*	*	*	*	-	*	6
Mijiritsky et al., (2009) [29]	*	*	*	*	*	*	*	7
Kher et al., (2018) [30]	*	*	*	*	*	-	*	6
Cosyn et al., (2016) [32]	*	*	*	*	*	*	*	7

*Characteristics of Included Studies* 

Eleven studies were included, out of which five were RCTs, four were retrospective studies, one was a descriptive study, and one was a prospective study. Overall, 2757 patients with caries were included for dental implants placed in the esthetic zone. The follow-up period ranged from one month to five years. Two studies recorded five dental failures [26,33] and three recorded zero dental failure [24,30,31]. The most mentioned sites were maxillary incisors, anterior maxilla, and canines (Table 2).

**Table 2:** Characteristics of the included studies SST: socket-shield technique, NR: not reported, RCT: randomized controlled trial

Author Name	Study Design	Participants	Techniques	Failure Rate	Survival Rate	Follow-Up	Reason for Extraction	Distribution of Sites Treated	Complications
Tiwari et al., (2019) [23]	RCT	16 patients aged 18-30 years	SST in the esthetic region & without SST	0/16	16/16	1 – 12 months	NR	Maxillary anterior region	Apical resorption of the shield
Sun et al., (2019) [24]	RCT	30 adult patients aged >25 years	SST & conventional flap-less immediate	0/30	30/30	1 – 24 months	Trauma & decay/pulp lesion	Incisor & canine	No complication
Siormpa, (2014) [25]	Retrospective	46 patients, 20 men & 26 women aged 28 – 70 years	Root-membrane technique	1/46	45/46	24 – 60 months	Extensive caries or supra-crustal horizontal fractures; cervical root resorption	Maxillary anterior teeth	Apical resorption
Gluckman et al., (2017) [26]	Retrospective	70 females, 58 males aged 24 – 71 years	SST & immediate implant placement	5/128 failed	123/128 survived	1-4 years	NR	Maxillary (incisors, canines, premolars) & mandibular (central incisors, canines, premolars)	Internal & external exposure, infection, implant failure, migration
Amir Alireza, (2015) [27]	Descriptive	2,381 Implants	Immediate/early/delayed implant placement in the esthetic region	0.8%	99.1%	2 years	NR	Maxillary & mandibular first premolar to first premolar	Implant failure, Dehiscence
De Rouck et al., (2008) [28]	Retrospective	30 patients, 14 males & 16 females, aged 24-76 years	immediate implant placement	1/30	29/30	1-12 months	Fracture, caries/endodontic, periodontal, root resorption	Anterior maxilla	Implant failure
Mijiritsky et al., (2009) [29]	RCT	24 implants, 16 patients, 7 females & 9 males, aged 23 – 62 years	Immediate implant placement	1/24	95.8%	Up to 6 years	Non-restorable crowns followed by root fractures	Maxillary esthetic zone	Implant failure
Kher et al., (2018) [30]	Retrospective	17 patients, 21 implants, 8 females	SST	0/21	100%	12–42 months	NR	Esthetic zone	Early shield exposure, midfacial recession
Bramanti et al., (2018) [31]	RCT	40 patients	SST technique & conventional	0/40	100%	1-36 months	Horizontal or vertical fracture, destructive caries, internal resorption, and endodontic problems not treatable with root canal therapy	Maxillary/mandibular teeth between canines	No complications
Cosyn et al., (2016) [32]	Prospective study	22 patients, 12 men & 10 women. Aged 27-74	Immediate implant placement	1/22	21/22	Up to 5 years	Root fracture, caries, root resorption	Central and lateral incisor, premolar, cuspid position	Implant failure, aesthetic, midfacial recession
Malo et al., (2003) [33]	Prospective clinical study	116 implants, 76 patients (41 males, 35 females) aged 18-81 years	Immediate and early implant placement	5/116	95.7%	1 year	NR	Maxillary teeth (74), mandibular teeth (42)	Fistulas, loss of suture gingival retraction, paresthesia, implant failure

Meta-Analysis of Dental Failure Rate

**Figure 3:**
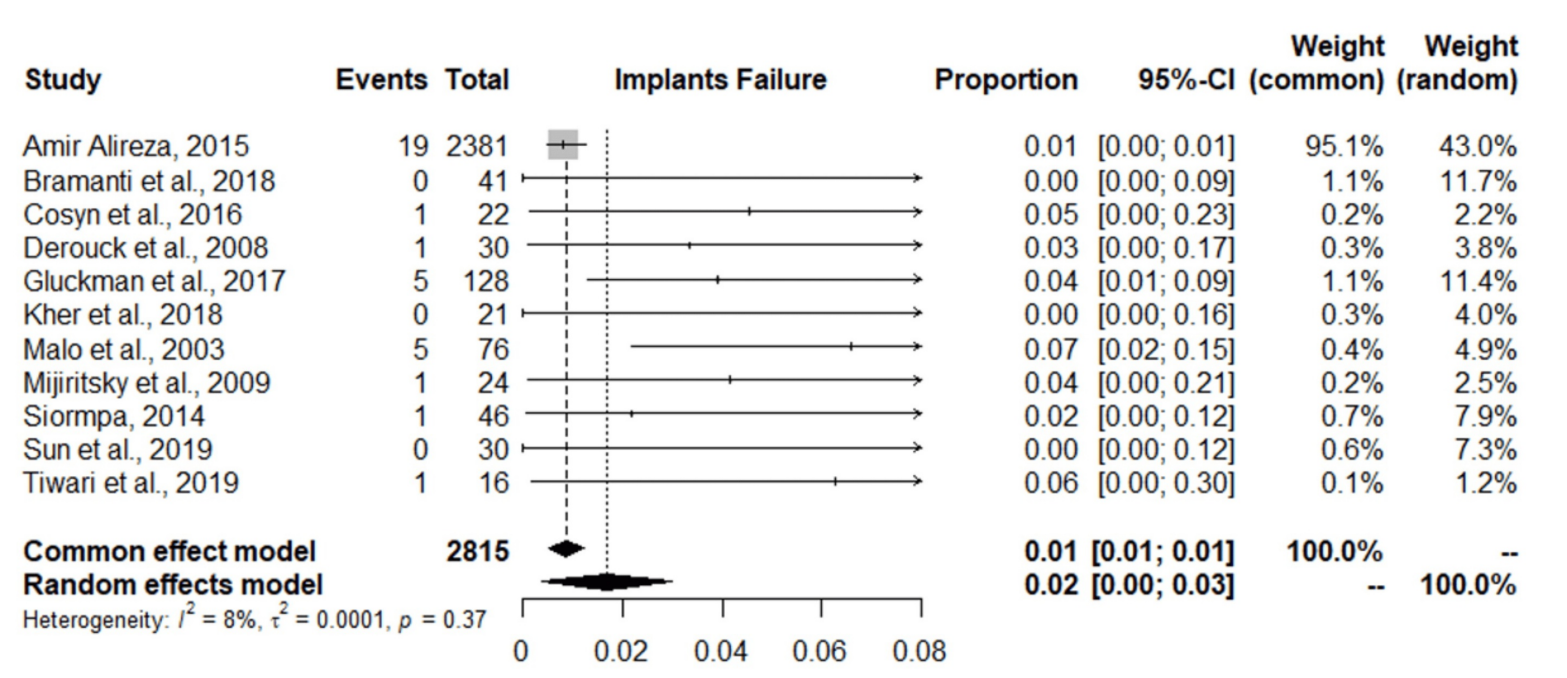
Forest plot of the meta-analysis of the failure rate of dental implants placed in the esthetic zone References [23-33]

**Figure 4:**
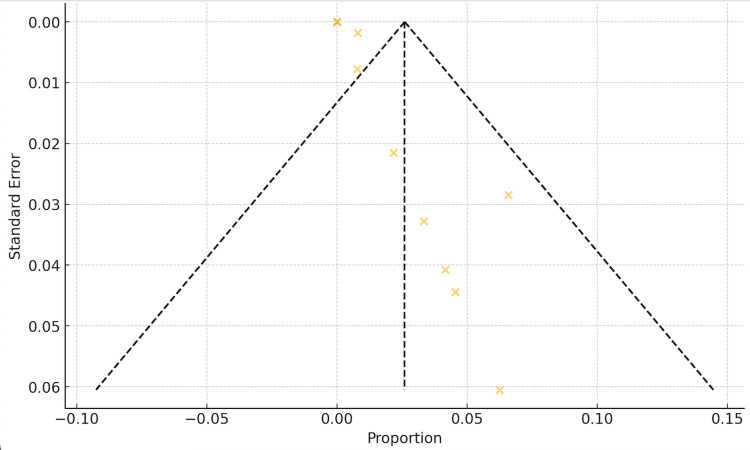
Funnel plot of studies included in the meta-analysis of the failure rate of implants placed in the esthetic zone References [23-33]

**Figure 5:**
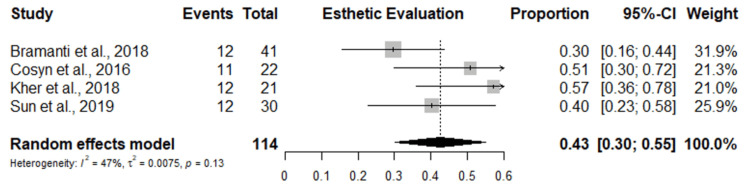
Forest plots of esthetic evaluation during the follow-up period References [24,30,31,32]

**Figure 6:**
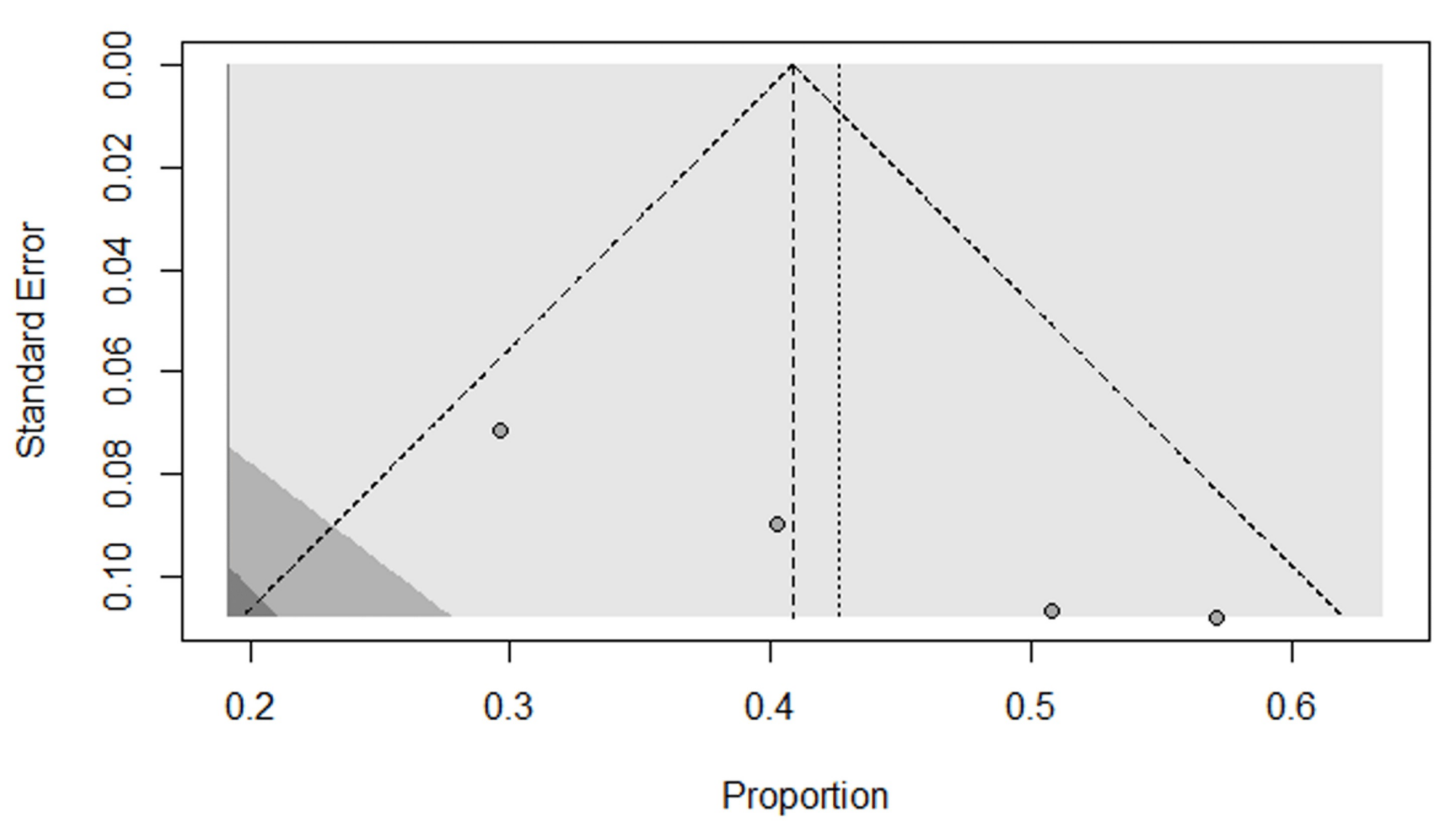
Funnel plot of studies included in the meta-analysis of esthetic evaluation during the follow-up period References [24,30-32]

Eleven studies were included in the meta-analysis of dental implant failure rate with a total of 2815 implants. The study with the highest dental failure is by Alireza et al. [27], followed by Gluckman et al. and Maló et al. [26,33] with five dental implants each. A random-effects model combined with a common-effects model was used to pool the studies. The results show an overall dental failure rate for dental implants placed in the esthetic zone of 2%, with a 95% confidence interval of 0.00-0.03%. Low insignificant heterogeneity was observed among the studies (I2 = 8%, p-value = 0.37, Q-test = 0.0001) (Figure 3). The funnel plot shows a symmetric shape implying that no evidence of publication bias was recorded (Figure 4). Figures 5-6 show the esthetic evaluation during follow-up periods.

Meta-Analysis of Marginal Bone Loss

**Figure 7:**
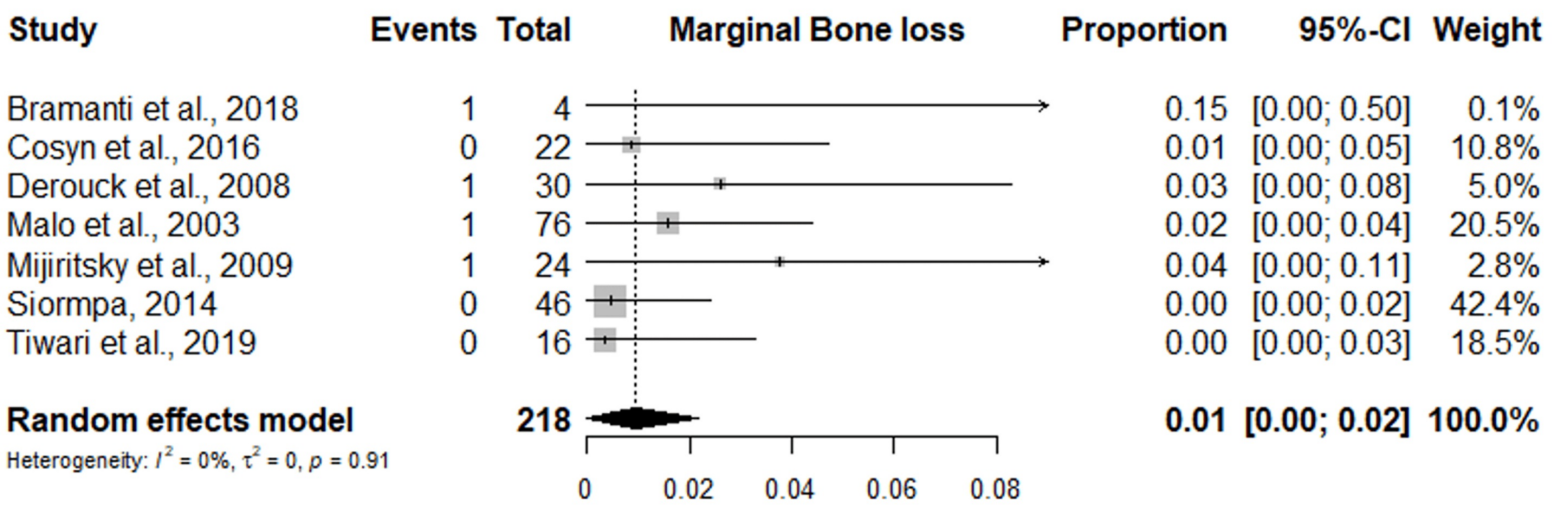
Forest plot of meta-analysis of Marginal bone loss in dental implants placed in esthetic zone. References [23,25,28,29,31-33]

Seven studies reported marginal bone loss during the dental implants placed in the esthetic zone. The random effect meta-analysis shows that the overall marginal bone loss was 1% with a 95% confidence interval of 0.00%-0.02%, with no heterogeneity among the studies (I2 = 0%) (Figure 7).

**Figure 8:**
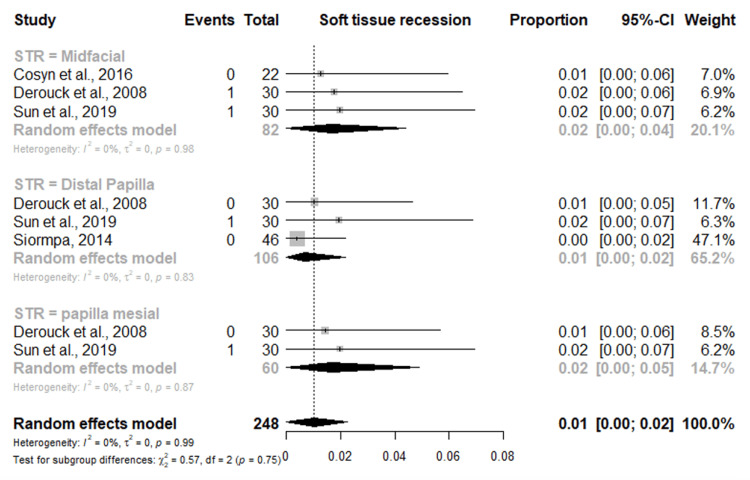
Forest plot of the meta-analysis of soft tissue recession outcomes in dental implants placed in the esthetic zone References [24,25,28,32]

The pink esthetic score showed an overall mean score of 11.75 and a proportion of 0.43%. The proportion of mid-facial recession recorded across all studies was 0.02%. The mesial and distal papillary recession was 0.02% and 0.01%, respectively (Figure 8). 

Discussion 

The present systematic review and meta-analysis has systematically reviewed and analyzed dental implant failure for implants placed in the esthetic zone. The results of the meta-analysis supported the results of previous studies that dental implants placed in the esthetic zone have a recorded success rate of 98%, implying a failure rate of approximately 2% [18,19].

Based on the present meta-analysis, the mean dental implant failure rate was 2% with a 95% confidence interval of 0.00-0.03% for the socket shield technique. Moreover, the study by Alireza et al. [27] had the highest sample size of 1281 implants and a reported success rate of 99.1% with just a 0.8% failure rate, and the study with the smallest sample size recorded a 100% success rate with a zero failure rate and the complication of apical resorption of the shield [23]. The results are slightly consistent with the previous literature that reported an overall failure rate of 1.37% but heterogeneity among the studies was not found [18], and one of the included studies with 76 dental implants with a one-year follow-up period had a success rate of 98.1% with a dental implant failure rate of 1.9%; however, the reported complications were fistulas, gingival retraction, and paresthesia [33].

According to the literature, the main function of the socket-shield technique for dental implants placed in the esthetic zone is to preserve the buccal bone plate that is capable of enhancing the esthetic outcome [17]; therefore, the distribution site reported in most of the included studies were placed in the anterior maxilla. However, the anterior maxillary site usually increases the risk of marginal bone resorption and apical resorption of the shield after dental implantation [34]. In this research, the overall marginal bone loss was 1% and no heterogeneity was found among the studies; moreover, Bramanti et al. [31] found no resorption of the root portions left in the site after the extraction because the fasciculate bone of the internal portion of the alveolus is typically reabsorbed after tooth extraction without the surrounding periodontal tissues, the thickness of the marginal bone crest around teeth can remain stable due to the vascular supply from the periodontal vessels, but a thin marginal bone crest around dental implants can resorb, exposing the rough surface of the implant [35].

As regards esthetic evaluation using the pink esthetic score, we found an overall mean score of 11.75 and a proportion of 0.43%. This measures the levels of soft-tissue recession for the socket-shied techniques compared to other conventional techniques. The estimated proportional aesthetic outcome showed the proportion of mid-facial recession recorded across all studies was 0.02% that of mesial and distal papillary recession was 0.02% and 0.01%, respectively. The socket-shield approach for immediate implant maintains the marginal bone crest, which may contribute to the high pink esthetic score reported in some of the included studies [24,30]. The pink esthetic score is frequently used to assess the pink esthetic around dental immediate implants since it was chosen as the most trustworthy and valid of the eight esthetic evaluation indices [36]. The PES measuring index takes into account the alveolar process deficiency, the soft tissue level and contour, the soft tissue color and texture, and the mesial and distal papilla insertion level. The few volumetric changes of the soft tissues and, consequently, the preserved marginal bone crest surrounding the immediate dental implants with the socket-shield technique may be responsible for the high mean PESs (11.75 (range, 11.12-12.61)) with a proportion of 0.43% found in this systematic review with meta-analysis.

Regarding the limitations encountered during the database search and the interpretation of results, the small sample size is due to the exclusion of a large number of studies, as only relevant studies were selected. In addition, low and insignificant heterogeneity found in the results was due to articles with multiple study designs and the same placement site. Therefore, future research is needed to validate this source of heterogeneity by adding studies.

## Conclusions

Dental implant-supported prostheses have significantly expanded prosthodontic options, enhancing patients' quality of life, especially when restoring missing teeth in the esthetic zone.

Based on this systematic review and meta-analysis, the rate of dental implant failure with implants placed in the esthetic zone either by the socket-shield technique or the conventional method was minimal. Reported complications included apical resorption, infection, dehiscence, fistulas, recession, and implant failure. In addition, 1% proportional marginal bone loss and moderately high esthetic scores were found. However, the risk of implant failure due to variables like smoking, bone quality, or systemic health must be carefully monitored.

In conclusion, a dental implant placed in the esthetic zone has shown a success rate of 98% with a failure rate of approximately 2%. Studies with larger sample sizes of implants placed in the esthetic zone are needed.
